# Labial gland-derived mesenchymal stem cells and their exosomes ameliorate murine Sjögren's syndrome by modulating the balance of Treg and Th17 cells

**DOI:** 10.1186/s13287-021-02541-0

**Published:** 2021-08-26

**Authors:** Boya Li, Yixiao Xing, Yehua Gan, Jing He, Hong Hua

**Affiliations:** 1 Department of Oral Medicine, Peking University School and Hospital of Stomatology, No. 22, Zhongguancun South Avenue, Haidian District, Beijing, 100081 People’s Republic of China; 2 Central Laboratory, Peking University School and Hospital of Stomatology, No. 22, Zhongguancun South Avenue, Haidian District, Beijing, 100081 People’s Republic of China; 3 Department of Rheumatology and Immunology, Peking University People’s Hospital, No. 11, Xizhimen South Street, Xicheng District, Beijing, 100044 People’s Republic of China

**Keywords:** Sjögren's syndrome, Autoimmune, Mesenchymal stem cells, Exosomes, T helper 17 cells, Regulatory T cells

## Abstract

**Background:**

Sjögren's syndrome (SS) is a chronic, systemic autoimmune disorder characterized by sicca syndrome and/or systemic manifestations. The disease severely affects the health and life of patients, and the treatment of SS has always been a clinical challenge and essentially palliative. Mesenchymal stem cells (MSCs) have been reported to exert immunomodulatory effects and as a potential novel therapeutic strategy for SS. Labial gland-derived MSCs (LGMSCs) are a population of resident stem cells in the labial gland, first isolated by our group. Exosomes released by MSCs contain a large variety of bioactive molecules and considered to function as an extension of MSCs.

**Methods:**

LGMSCs were isolated from patients who were needed surgery to remove the lip mucocele and LGMSCs derived exosomes (LGMSC-Exos) were isolated by ultracentrifugation. The non-obese diabetic (NOD) mice were treated with LGMSCs or LGMSC-Exos by tail vein injection. The saliva flow rate of mice was determined and salivary glands were dissected and stained with hematoxylin and eosin. In vitro, peripheral blood mononuclear cells (PBMCs) from SS patients were cocultured with LGMSCs or LGMSC-Exos. Percentage of T helper 17 (Th17) cells and regulatory T (Treg) cells were determined by flow cytometry. The serum levels of cytokines in NOD mice and in the supernatant of the co-culture system by ELISA.

**Results:**

Treatment with LGMSCs or LGMSC-Exos reduced inflammatory infiltration in the salivary glands, and restored salivary gland secretory function in NOD mice. Importantly, LGMSCs or LGMSC-Exos were demonstrated to inhibit the differentiation of Th17 cells but promote the induction of Treg cells in NOD mice and PBMCs from SS patients in vitro, accompanied by reduced interleukin 17 (IL-17), interferon gamma, and IL-6 levels and enhanced transforming growth factor beta and IL-10 secretion by T cells.

**Conclusions:**

LGMSCs are potential candidates for MSCs-based therapy and LGMSC-Exos might be utilized for establishing a new cell-free therapy against SS.

**Supplementary Information:**

The online version contains supplementary material available at 10.1186/s13287-021-02541-0.

## Introduction

Sjögren’s syndrome (SS) is a chronic systemic rheumatic disease characterized by lymphoplasmacytic infiltration into the exocrine glands, including salivary and lacrimal glands, and results in progressive loss of secretory function [1, 2]. SS is the most frequent connective tissue disease after rheumatoid arthritis (RA) and is characterized by a high sex preponderance with the ratio of nine females to one male. Moreover, the incidence of SS peaks at approximately 50 years of age. The commonly accepted estimated prevalence of the disease is 0.51% in the general population. The incidence rate of SS is 3.9 to 6.92 per 100,000 person-years according to the different studies [3].

The hallmark characteristics of SS are dryness of the mouth and eyes, fatigue, and joint pain, and the disease is associated with a wide spectrum of systemic manifestations [4]. The disease can be encountered alone (primary Sjögren’s syndrome) or in the presence of another autoimmune disorder such as systemic lupus erythematosus (SLE), RA, or scleroderma. In addition to sicca syndrome due to chronic inflammation of glandular tissue, systemic manifestations occur in 30%–40% of patients with pSS, and the risk of B-cell lymphoma is markedly increased by 15–20 times among these patients when compared with that in the general population [4]. Thus, SS has a severe influence on patients’ quality of life, and increases mortality risk among the patients. In summary, the widespread prevalence, the diverse clinical picture of the disease, and the coexistence with lymphoma conferring to the patients’ morbidity and mortality creates an urgency to establish effective therapeutic strategies for SS.

So far, the therapeutic management of SS has not changed substantially in recent decades and is still based on symptomatic and alternative treatment of sicca symptoms, as well as broad-spectrum immunosuppressants and glucocorticoids for abnormal autoimmune reactions, with insufficient information available on the differential efficacy and safety of the therapeutic options [5]. Thus, a safer and more effective therapy that protects against SS is urgently needed.

SS is a chronic autoimmune disorder that is mediated by T and B lymphocytes. Although the overproduced auto-antibodies are secreted by B cells, T cells are the major infiltrators in most phases of the disease, and the activation of T cells leads to tissue damage and secretory dysfunction [6]. An imbalance between type 1 cytokine-producing T helper 1 (Th1) cells and type 2 cytokine-producing Th2 cells has been considered to predispose SS to autoimmunity. In the last decade, a number of Th cell lineages have been identified, including Th17, regulatory T (Treg), and follicular helper T cells [7]. Tregs actively suppress pathological and physiological immune responses, maintain immunological tolerance, and prevent autoimmunity [8]. Unregulated Th17 responses or excessive interleukin-17 (IL-17) production from T cells and other sources is associated with chronic inflammation and severe immunopathological conditions [9]. Currently, the data available suggest a pivotal role of IL-17 axis and Th17 cells in the pathogenesis of SS [10] and highlight a possible shift in the Th17/Treg balance towards proinflammatory Th17 cells [11].

In recent years, mesenchymal stem cells (MSCs) have emerged as a promising tool for treating SS, owing to their low immunogenicity and immunosuppressive properties [12]. MSC transplantation has been extensively investigated, and accumulating evidence suggests that MSCs from different sources are all effective in the treatment of SS [13–16]. Labial gland-derived MSCs (LGMSCs) are a type of MSC that were first isolated by our group and meet the criteria for MSCs [17]. Labial minor salivary glands have a superficial location, are easy to obtain, and are widely used for tissue sampling in gland biopsies. The abundant and easily accessible features of LGMSCs made them a kind of ideal MSCs for stem cells-based therapy and we aimed to evaluate the potential therapeutic role of LG-MSCs in treating SS.

Extracellular vesicles (EVs) are phospholipid bilayer structures that are secreted by almost all cell types. EVs with a diameter of 40–160 nm (average ~ 100 nm), which are produced by exocytosis of multivesicular endosomes are called exosomes [18]. By transferring functional molecules between cells, exosomes act as potent mediators of intercellular communication and play significant roles in various biological functions [19, 20]. Recent evidence suggests that the immunomodulatory effects of MSCs are at least partially mediated by secreted microvesicles [21], indicating that MSC-derived EVs exhibit immunomodulatory effects [22, 23]. Recent experiments have indicated a function of exosomes in eliciting adaptive and innate immune reactions, supporting their utility for therapy development and a potential role in coordinating immune reactions [24]. Therefore, elucidating the effect of LGMSCs and LGMSC-derived exosomes (LGMSC-Exos) on T cells in SS is noteworthy.

In this study, we investigated the feasibility of LGMSCs and LGMSC-Exos as immunosuppressants in the treatment of SS, and their possible mechanisms, with a special emphasis on Th17 and Treg cells, were explored in both experimental animal models and in vitro culture of peripheral blood mononuclear cells (PBMCs) obtained from SS patients.

## Materials and methods

### Mice

Female non-obese diabetic (NOD) mice and BALB/c mice aged 6–8-week-old were purchased from the Experimental Animal Center of Peking Medical University and maintained in a specific pathogen-free environment. The mice were fed standard rodent chow and water ad libitum. The Peking University Institutional Review Board for the care and use of laboratory animals approved all the experiments in this study (LA2019134).

### Isolation, culture, and in vitro characterization of human LGMSCs

#### Isolation of human LGMSCs

This study was approved by the institutional review board of Peking University School of Stomatology (PKUSSIRB-201631139). Five patients (basic information of these five donors showed in Additional file 1: Table S1) did not have any systemic diseases and needed surgery to remove the lip mucocele were informed about the research project, and informed consent forms were signed. After enucleation of lip mucoceles, the surrounding normal labial gland tissues were isolated and washed with phosphate-buffered saline (PBS). Fibrous tissues, capsules, and blood vessels around the labial glands were removed, and the remaining tissue was cut into small pieces using ophthalmic scissors, placed in cell culture flasks, and maintained in alpha minimum essential Eagle’s medium (α-MEM) (Gibco, Waltham, MA, USA) supplemented with 10% fetal bovine serum (FBS) (Gibco) and 100 U/mL penicillin/streptomycin (Beyotime, Shanghai, China) at 37 ℃ in 5% CO_2_. The medium was refreshed every 3 days. After reaching 80% confluence, the cells were detached with 0.05% trypsin/EDTA (Gibco) and passaged. All experiments were performed using cells between passages three and six.

#### Characterization of LGMSCs

For phenotype analysis, third-generation LGMSCs were digested, harvested, and washed with cold PBS. The cells were then labeled with PE-conjugated antibodies against CD29, CD73, CD31, CD45 and isotype control IgG1 or labeled with PE-conjugated antibodies against CD80, CD86, HLA-DR and isotype control IgG1 and IgG2a (BD biosciences, Franklin Lake, NJ, USA) (for immunogenicity analysis) for 30 min in the dark at 4 ℃. The cells were washed twice with stain buffer and analyzed using flow cytometry.

To evaluate the multilineage differentiation capacity, LGMSCs were cultured in the corresponding differentiation medium after reaching 80% confluence. The osteogenic induction medium consisted of α-MEM supplemented with 10% FBS, 10 nM dexamethasone (Sigma-Aldrich, St Louis, MO, USA), 0.2 mM ascorbic acid (Sigma-Aldrich), and 10 mM β-glycerophosphate disodium salt hydrate (Sigma-Aldrich). The medium was refreshed every 3 days. After 3 weeks, alizarin red (Sigma-Aldrich) staining was used to detect the formation of mineralized nodules. The adipogenic induction medium consisted of α-MEM supplemented with 10% FBS, 0.5 mM 3-isobutyl-1-methylxanthine (Sigma-Aldrich), 2 μM dexamethasone, 0.2 mM indomethacin (Sigma-Aldrich), and 0.01 g/L insulin (Sigma-Aldrich). After 2 weeks, the cells were stained with oil red O (Sigma-Aldrich).

### Isolation and characterization of LGMSC-Exos

#### Isolation of LGMSC-Exos

After LGMSCs were cultured in α-MEM with exosome-depleted FBS for 48 h, the supernatants were collected and centrifuged at 300 × g for 10 min, 2000 × g for 10 min, and 10,000 × g for 30 min. Then the supernatants were ultracentrifuged at 100,000 × g for 60 min at 4 ℃ using ultracentrifugation by Optima L-100XP ultracentrifuge (Beckman Coulter, Placentia, CA, USA). After washed with PBS at 100,000 × g for another 60 min at 4 °C, the purified exosomes were resuspended in PBS and quantified using a Micro-BCA protein assay kit (Thermo Fisher Scientific, Waltham, MA, USA). The fractions were stored − 80 ℃ until use. Exosomes were characterized via transmission electron microscopy (TEM), nanoparticle tracking analysis (NTA), and exosomal marker expression analysis.

#### Electron microscopy

TEM was used to characterize isolated exosomes. The LGMSC-Exos samples were diluted with PBS to a concentration of about 10^11^ vesicles per milliliter and then add 10 μL of diluted sample onto the carbon film supported copper grid for 5 min in room temperature. After drying the sample, adding 10μ L phosphotungstic acid to the copper grid and stain for 1 min. The excess liquid was sucked up with filter paper and the sample was dried at room temperature before observed under TEM and photographed.

#### NTA

The particle size of LGMSC-Exos was analyzed via NTA using a ZetaView PMX 110 (Particle Metrix, Munich, Germany) and analyzed by the software ZetaView. Exosomes were diluted in PBS over a range of concentrations between 2 × 10^8^ and 8 × 10^8^ vesicles per milliliter. Then the samples were introduced into the sample chamber for video recording. The ZetaView system was calibrated using 110 nm polystyrene particles and NTA settings were optimized and kept constant between samples. The samples were advanced between each recording to perform replicate measurements and each video was analyzed to give the mean, mode, and median vesicle size together with an estimate of the concentration.

#### Western blotting

To confirm the purified pellets as exosomes, markers of exosomes, including CD63, CD9, and CD81, were detected via western blotting. The pellets were separated using sodium dodecyl sulfate–polyacrylamide gel electrophoresis and transferred onto polyvinylidene difluoride membranes. The membranes were subsequently blocked with 5% nonfat-dried milk solution at room temperature for 1 h and incubated with anti-human CD9, CD63, and CD81 (1:1000 dilution) (Abcam, Cambridge, UK) overnight at 4 ℃. The membranes were then washed and incubated with secondary antibodies conjugated with horseradish peroxidase for 1 h at room temperature. Finally, the bands were observed by enhanced chemiluminescence (Millipore, Billerica, MA, USA) using an X-ray film.

### Assessment the effectiveness of allogeneic LGMSCs and LGMSCs-Exos treatment on NOD mice

#### Mice treatment

For human LGMSCs and LGMSC-Exos treatment, 8-week-old female NOD mice were injected with LGMSCs (10^6^ cells/mouse) or LGMSC-Exos (50 μg/mouse) into the tail vein on alternate days for two weeks. BMMSC-injected and hydroxychloroquine (HCQ) gastric infused mice served as positive controls [25, 26], and the mouse injected with an equal volume of PBS served as a negative control. BMMSCs (10^6^ cells/mouse) were injected into the tail vein on alternate days for two weeks. For HCQ group, the mice were intragastrically administrated with 0.4 ml HCQ dilution per day in accordance with 60 mg/kg dose for 6 weeks. The blood glucose levels of NOD mice were monitored weekly from 12 weeks of age.

#### Saliva flow rate measurement

The saliva flow rate of mice was determined before, during, and after the treatment, as per a previous study [27]. After weighing the mice, they were anesthetized and injected with pilocarpine hydrochloride (0.1 mg/kg i.p.) to stimulate salivation. Saliva was collected 5 min after the pilocarpine injection as follows: a glass capillary was placed on the side of the mouth under the tongue and held steadily during a 10-min period to collect saliva into a microcentrifuge tube (Additional file 1: Figure 1). The weight difference in the microcentrifuge tubes before and after saliva collection was calculated.

#### Histological analysis of salivary glands

Salivary and lacrimal glands were dissected immediately after euthanizing the mice and fixed in 10% buffered formalin for 24 h. After dehydration and embedment, the tissues were sectioned at 5 μm and stained with hematoxylin and eosin (H&E) according to a standard histopathology protocol to determine the infiltration of lymphocytes. Infiltrates appear as periductal and perivascular foci within the glandular structure of the salivary glands. Histological evaluation was performed in a blinded manner and scored according to the method proposed by Ishimaru et al. [28].

#### Detection of apoptosis of salivary gland cells

One-step TUNEL Cell Apoptosis Assay Kit (KeyGEN BioTECH, Jiangsu, China) were used to visualize apoptotic cells in salivary glands of NOD mice after different treatment. Paraffin sections were processed according to the manufacturer’s instructions. Nuclei were visualized with DAPI (blue) and apoptotic cells were visualized with TRITC (red). Two blind examiners independently counted the absolute number of apoptotic cells in three random fields per tissue section under 400 × magnification. At least three random tissue sections per gland were chosen for each slide.

### Co-culture of LGMSCs and LGMSC-Exos with PBMCs from SS patient

Human PBMCs were isolated from SS patients and healthy controls via density sedimentation on Ficoll gradients. PBMCs were cultured in complete RPMI 1640 medium (Gibco) supplemented with CD3/CD28 Dynabeads (Biolgend). The ratio of LGMSCs to PBMCs was 1:10, and 30 μg/mL exosomes was used for the treatment. Seventy-two hours after the co-culture, PBMCs and the supernatant were collected for use in subsequent experiments. To examine the uptake of exosomes by PBMCs in vitro, LGMSCs were labeled using a PKH26 red fluorescent labeling kit (Sigma-Aldrich) and incubated with PBMCs for 12 h. The exosome-treated cells were then fixed and imaged.

### Flow cytometric analysis

Flow cytometric analysis was performed to detect CD4 + T cell subsets. Treg cell staining was performed following the FoxP3/Transcription Factor Buffer Set (eBioscience, San Diego, CA, USA) protocols and using anti-CD4, anti-CD25, and anti-FoxP3 mAbs (BioLegend, San Diego, CA, USA). Similarly, anti-CD4 and anti-IL17A mAbs (BioLegend) were used for Th17 cell staining as per the Intracellular Fixation and Permeabilization Buffer Set (eBioscience) protocols, after stimulating the cells with phorbol 12-myristate 13-acetate (50 ng/mL), ionomycin calcium (1 mg/mL), and brefeldin A (2 mg/mL) for 5 h. The cells were then analyzed using flow cytometry (FACS Calibur, Becton Dickinson, Franklin Lakes, NJ).

### Enzyme-linked immunosorbent assay (ELISA)

The serum levels of cytokines in NOD mice [IL-17A, IL-6, and transforming growth factor beta (TGF-β)] and in the supernatant of the co-culture system [human IL-10, IL-17A, TGF-β, interferon gamma (IFN-γ), and tumor necrosis factor alpha (TNF-α)] were determined using ELISA kits (BioLegend) according to the manufacturer’s protocol.

### Statistical analysis

Quantitative data are presented as mean ± standard deviation (SD). For normal distribution data, the statistical significance was determined via the Student’s *t*-test for comparison of two groups or one-way analysis of variance adjusted by the Bonferroni method for multiple comparisons. For non-normal distribution data, the Mann–Whitney U test or Kruskal comparison test were used. All analyses were performed using SPSS version 19.0. Statistical significance was set at *p* < 0.05.

## Results

### Isolation, culture, and identification of LGMSCs

**Fig. 1:**
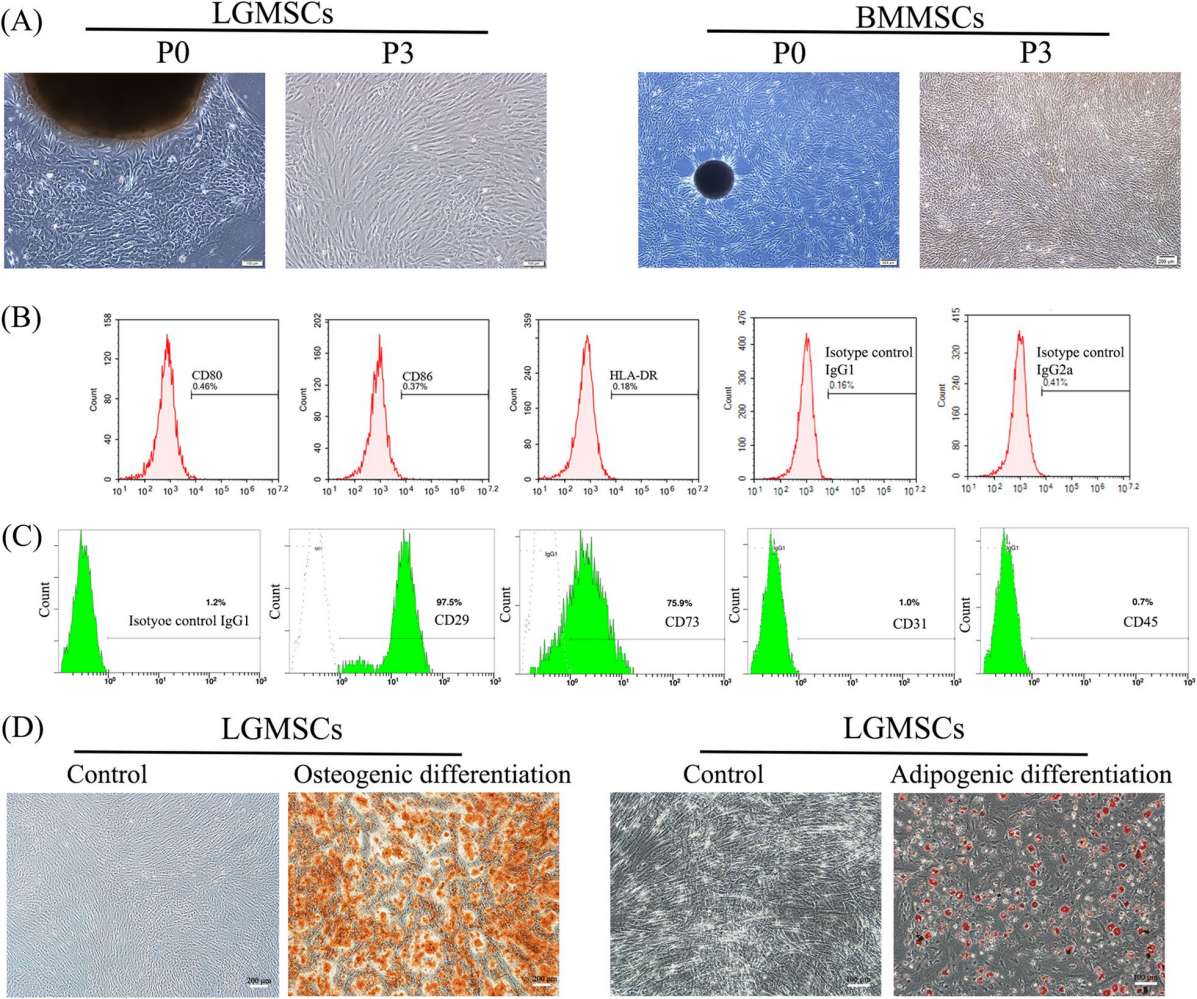
Isolation and identification of LGMSCs. **A** Human LGMSCs and BMMSCs at primary passage (P0) and passage 3 (P3). Scale bar: 200 μm. (B–C) Expression of surface costimulatory molecules **B** and other cell surface markers **C** on LGMSCs, determined via flow cytometry. **D** Alizarin red (scale bar: 200 μm) and oil red O staining (scale bar: 100 μm) of LGMSCs after inducing cell differentiation for 2 or 3 weeks proved their multipotentiality. *LGMSCs* labial gland-derived mesenchymal stem cells; *BMMSCs* bone marrow mesenchymal stem cells

Human LGMSCs were isolated and observed under an inverted phase-contrast microscope. Primary cells were successfully obtained and passaged stably. The shapes of LGMSCs were similar to those of fibroblasts and the cells were spirally arranged. There was no difference in morphology between LGMSCs and BMMSCs (Fig. 1A).

The immunogenicity of LGMSCs was detected via flow cytometry, and the results showed that the expression of surface costimulatory molecules, namely, CD80, CD86, and HLA-DR was all low or not expressed (Fig. 1B), indicating low immunogenicity of LGMSCs. Additionally, the expression of specific surface markers was estimated, and the results showed that LGMSCs were positive for MSC-specific surface markers (CD29 and CD73) but negative for hematopoietic and endothelial cell-specific markers (CD31 and CD45) (Fig. 1C). After osteogenic or adipogenic induction for 3 weeks, alizarin red-positive nodules and oil red O-positive lipid droplets were observed (Fig. 1D), which confirmed the multipotentiality of isolated cells. Overall, the data suggest that LGMSCs display typical characteristics of MSCs and have been successfully purified.

### Isolation and identification of exosomes derived from LGMSCs

**Fig. 2:**
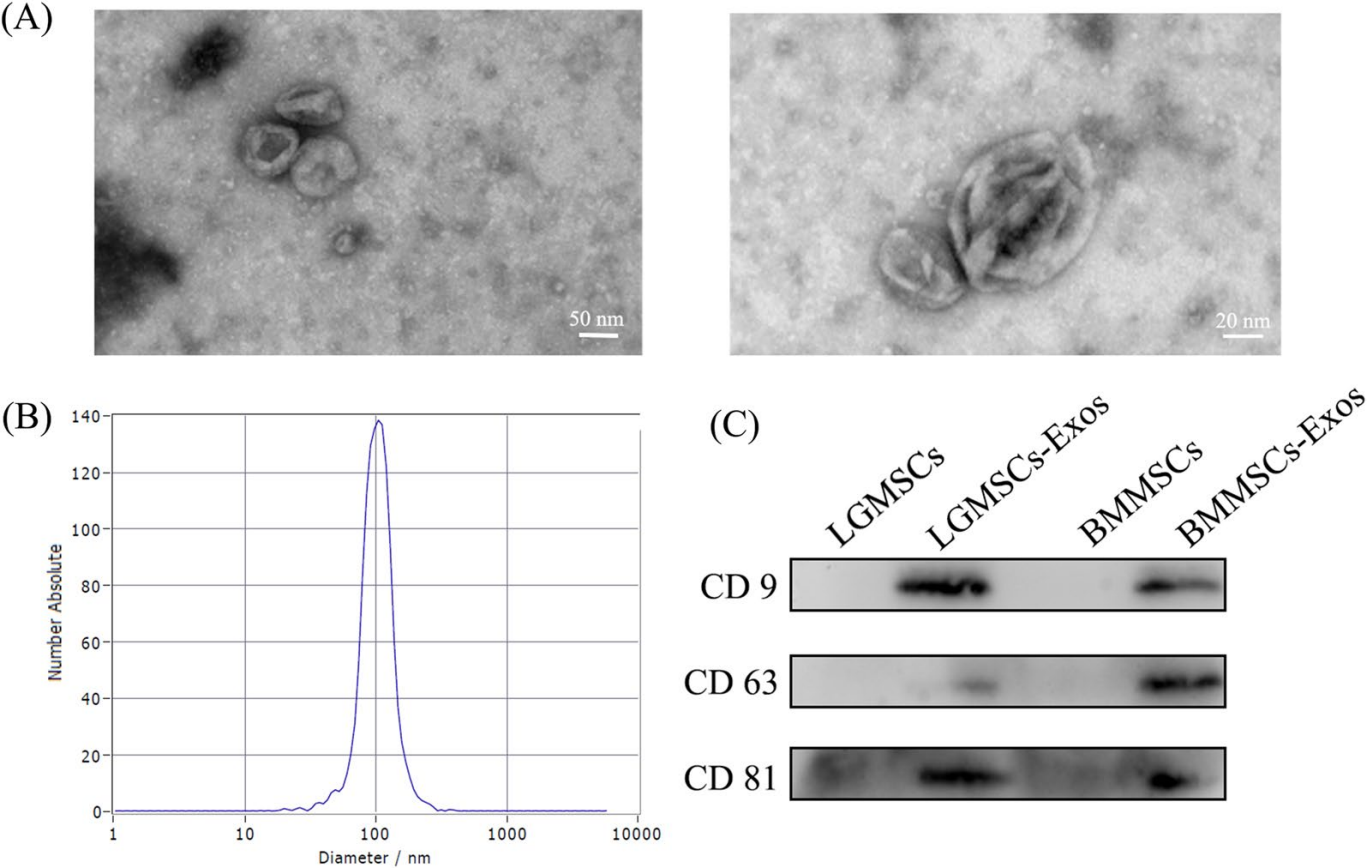
Isolation and identification of exosomes released from LGMSCs (LGMSC-Exos). **A** Representative TEM images of LGMSC-Exos. **B** NTA analysis of LGMSC-Exos. **C** Expression of specific markers of LGMSC-Exos (CD9, CD 63, and CD81) was analyzed via western blotting. *LGMSCs* labial gland-derived mesenchymal stem cells; *TEM* transmission electron microscopy; *NTA* nanoparticle tracking analysis

Exosomes were isolated from with supernatant of LGMSCs by ultracentrifugation. As shown in Fig. 2A, TEM micrographs exosomes from LGMSCs displayed typical cup-shaped. The size distribution was showed by NTA (Fig. 2B) and consistent with the exosomes. Western blot analysis showed the isolated exosome expressed CD9, CD63 and CD81 (Fig. 2C), which are typical markers of exosomes. Together, these data indicate the successful isolation and purification of LGMSCs-Exos.

### Treatment with LGMSCs and LGMSC-Exos alleviated SS-like disease in NOD mice.

**Fig. 3:**
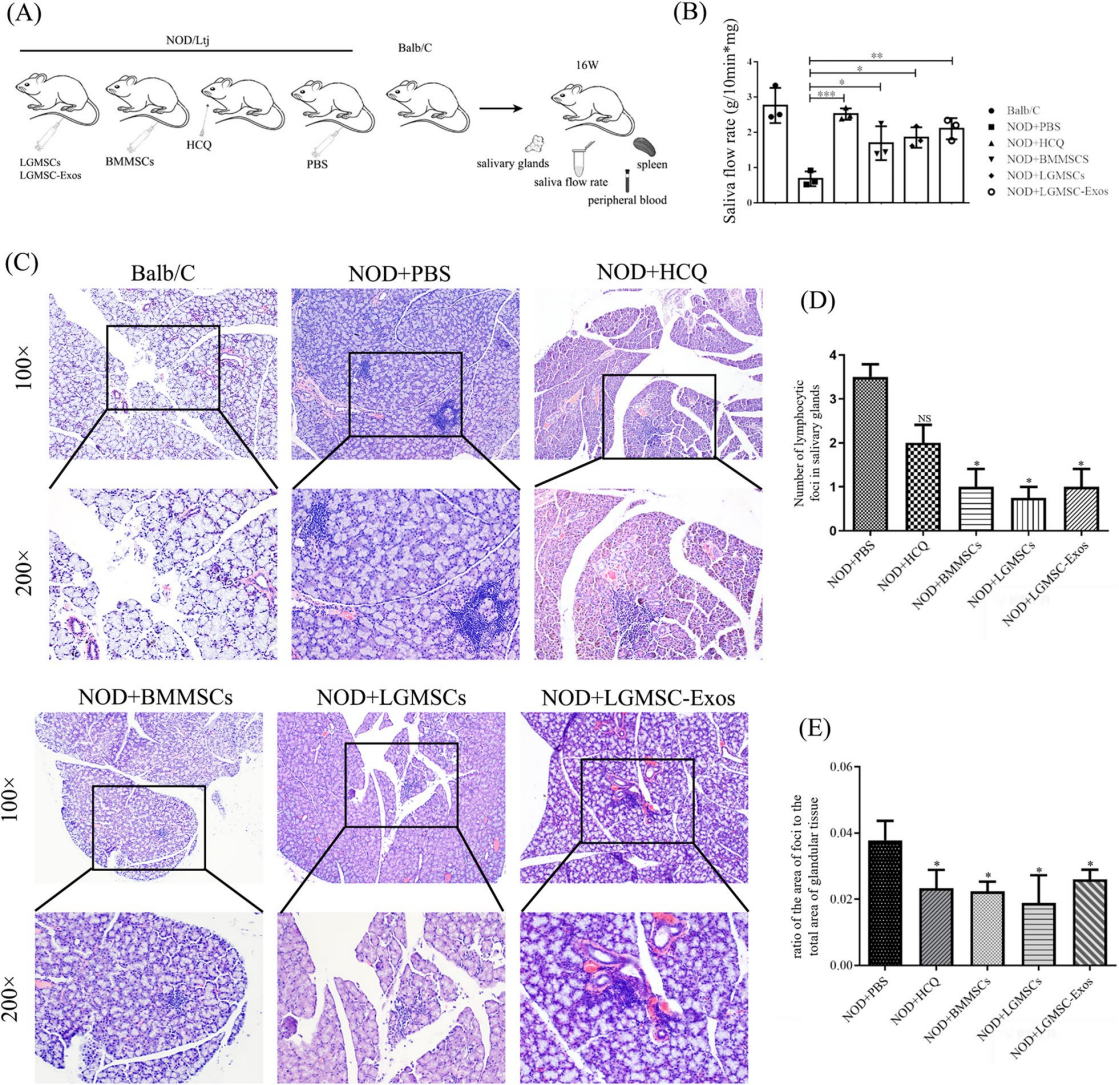
LGMSCs and LGMSC-Exos treatment reduced inflammatory infiltration and improved saliva flow rate in NOD mice. **A** Grouping and treatment of mice. **B** The saliva flow rate of mice in different groups. **C** Histology of the salivary glands of mice in different groups. Data are shown as mean ± standard deviation (SD) from three independent experiments. ***p < 0.001, **p < 0.01, *p < 0.05. **D** Statistical results of the number of inflammatory foci. **E** Statistical results of the ratio of the area of foci to the total area of glandular tissue. *p < 0.05 compared with NOD + PBS group. *HCQ* hydroxychloroquine; *LGMSCs* labial gland-derived mesenchymal stem cells; *LGMSC-Exos* LGMSC-derived exosomes

The mice were treated with LGMSCs and LGMSC-Exos by injecting the molecules into their tail vein. Female BALB/c mice (same weeks of age as NOD mice) were healthy controls (Fig. 3A). The blood glucose level of all the NOD mice were normal during the experiment (Additional file 1: Figure S2). After the treatment, the saliva flow rate of 16-week-old mice in each group was analyzed to evaluate the efficacy of the treatment. LGMSC- and exosome-treated NOD mice exhibited a higher rate of salivary flow than that of PBS-treated mice (*p* < 0.05, Fig. 3B). LGMSC- and LGMSC-Exos treatment showed similar protective effects as the positive controls (*p* > 0.05, Fig. 3B). H&E staining showed that the number and area of lymphocyte infiltration foci in salivary glands of LGMSC- and exosome-treated mice were considerable reduced than those in PBS-treated NOD mice (*p* < 0.05, Fig. 3C-E).

### Treatment with LGMSCs and LGMSC-Exos favored Treg responses but suppressed Th17 responses in NOD mice

**Fig. 4:**
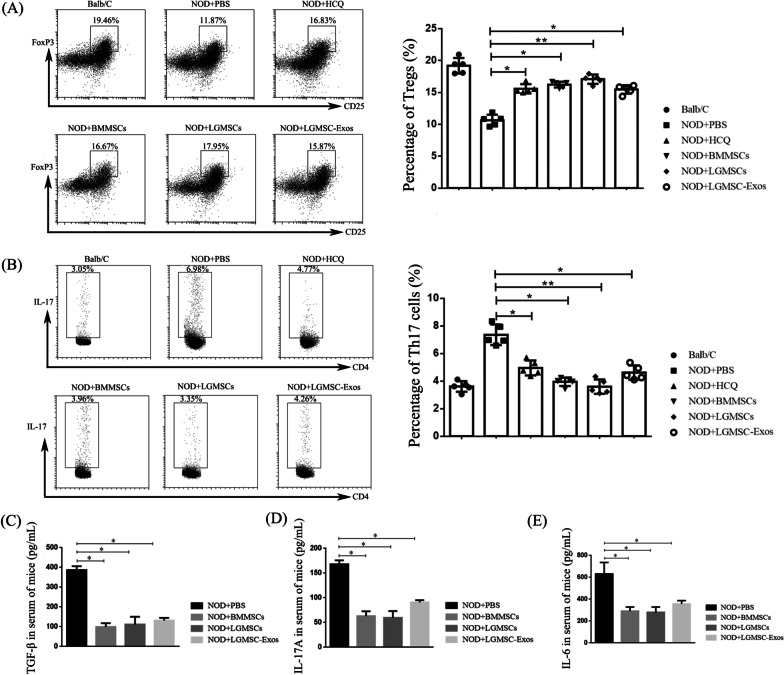
LGMSCs and LGMSC-Exos reduce Th17 cells and expand Tregs in NOD mice. **A**, **B** Proportions of Tregs (**A**) and Th17 cells (**B**) in spleen cells of NOD mice after different treatments. **C**–**E** Serum levels of TGF-β (**C**), IL-17 (**D**), and IL-6 (**E**) in NOD mice. Data are shown as mean ± standard deviation (SD) from three independent experiments. ***p < 0.001, **p < 0.01, *p < 0.05. *HCQ* hydroxychloroquine; *LGMSCs* labial gland-derived mesenchymal stem cells; *LGMSC-Exos* LGMSC-derived exosomes; *Th17* T helper 17; *Treg* T regulatory; *TGF-β* transforming growth factor beta; *IL* interleukin

The population of Treg and Th17 cells in the spleen of NOD mice was analyzed using flow cytometry. The number of CD4^+^ CD25^+^ Foxp3^+^ Tregs in the LGMSC- and LGMSC-Exo-treated mice was significantly higher than that in the PBS-treated controls (*p* < 0.05) (Fig. 4A). In addition, we examined the proportion of Th17 cells and showed that the proportion of this cell subset decreased significantly after treatment with LGMSCs and LGMSC-Exos (Fig. 4B).

As TGF-β and IL-10 are important for Treg cell proliferation and function, we estimated the serum levels of TGF-β and IL-10 and found that TGF-β levels in the serum of NOD mice increased after MSC and exosome infusion (Fig. 4C), whereas the serum levels of IL-10 did not change significantly (data not shown). The proinflammatory cytokine IL-17 activates a positive feedback loop that commits naive T cells to the Th17 lineage, and the results showed that the serum levels of IL-17 in NOD mice were significantly reduced after the treatment (Fig. 4D). Moreover, IL-6 participates in driving Th17 differentiation with or without TGF-β, and the results showed that the serum levels of IL-6 were reduced in MSC- and exosome-treated groups compared to those in PBS-treated controls (Fig. 4E).

### The proportion of Th17 and Treg cells was disturbed in the peripheral blood of SS patients

**Fig. 5:**
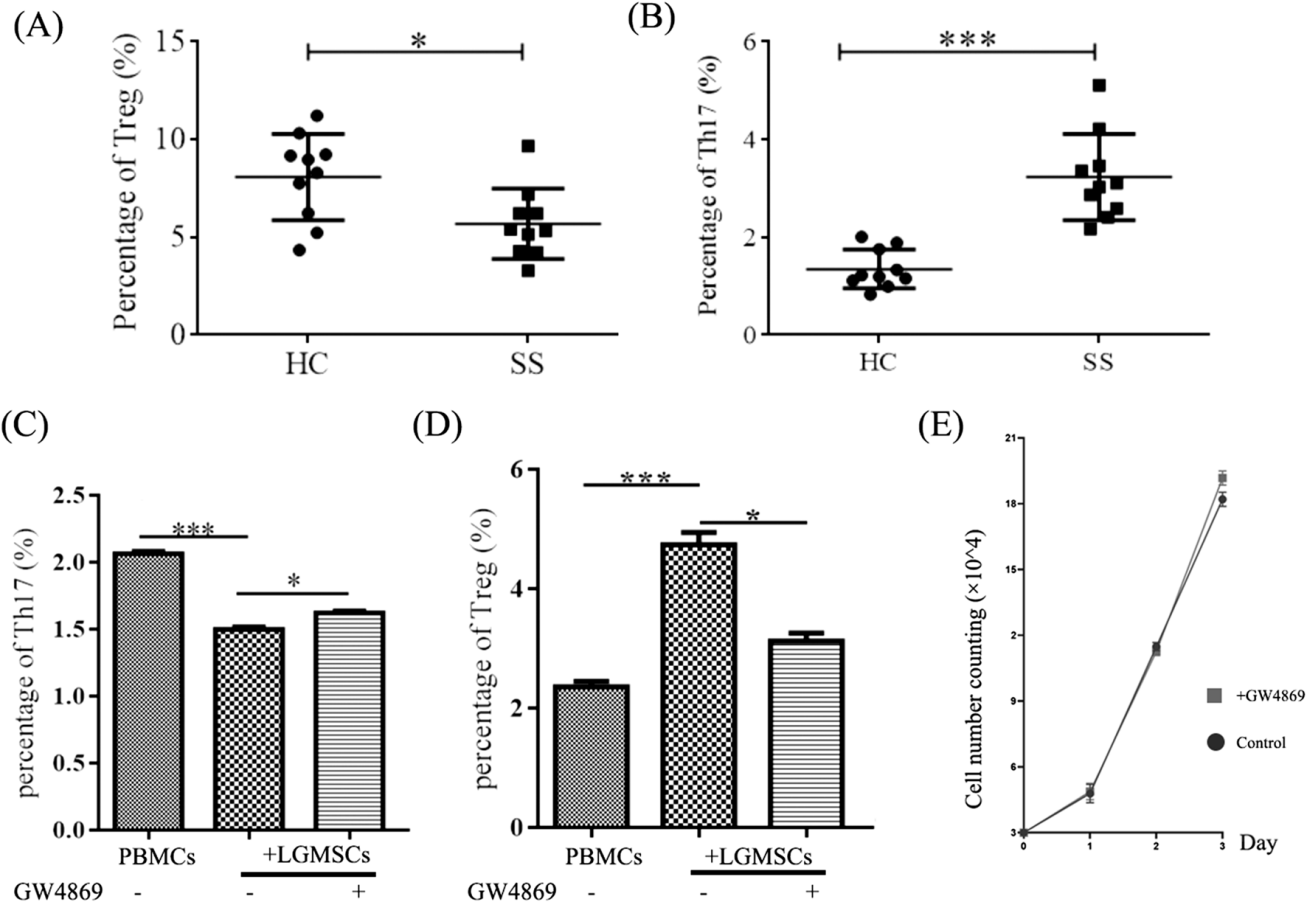
The proportion of Th17 and Treg cells. **A**, **B** The proportion of Treg (**A**) and Th17 (**B**) cells in PBMCs of systemic healthy donors and SS patients. **C**, **D** The proportion of Th17 cells (**C**) and Treg cells (**D**) in PBMCs from SS patients co-cultured with LGMSCs and GW4869-treated LGMSCs. **E** Cell number counting of LGMSCs and GW4869-treated LGMSCs. Data are shown as mean ± standard deviation (SD) from three independent experiments. ***p < 0.001, **p < 0.01, *p < 0.05. *Th17* T helper 17; *Treg* T regulatory; *PBMCs* peripheral blood mononuclear lymphocytes; *SS* Sjögren's syndrome; *LGMSCs* labial gland-derived mesenchymal stem cells

To confirm the shift in the Th17/Treg balance towards proinflammatory Th17 cells in SS patients, PBMCs from 10 SS patients and 10 matched controls were collected and the percentage of Th17 and Treg cell subsets was detected. The proportion of Th17 cells in the peripheral blood of SS patients was significantly increased (*p* < 0.01), whereas that of Treg cells was slightly decreased (Fig. 5A).

### LGMSCs and LGMSC-Exos displayed potent immunosuppressive effects by regulating Th17/Treg balance in SS patients in vitro

#### Effects of LGMSCs on Tregs and Th17 were dependent on secreted exosomes

PBMCs from patients with SS were isolated to further verify the results in NOD/Ltj mice. GW4869 is a commonly used pharmacological agent that inhibits exosome generation. It blocks ceramide-mediated inward budding of multivesicular bodies (MVBs) and the release of mature exosomes from MVBs. LGMSCs were pretreated with GW4869 (10 nM) to evaluate whether the effect of LGMSCs on CD4^+^ T cells was partially mediated by exosomes. Flow cytometry analysis results showed that GW4869 pretreatment partially reversed the effect of LGMSCs on the decrease in Th17 number and the increase in Treg cells (Fig. 5C, D). In addition, cell counting analysis indicated that GW4869 pretreatment did not affect the proliferation of LGMSCs (Fig. 5E), indirectly proving that the immunoregulatory activity of LGMSCs on CD4^+^ T cells was partially dependent on secreted exosomes.

#### LGMSCs and LGMSC-Exos suppressed Th17 cells and induced Treg cell proliferation in SS patients in vitro

**Fig. 6:**
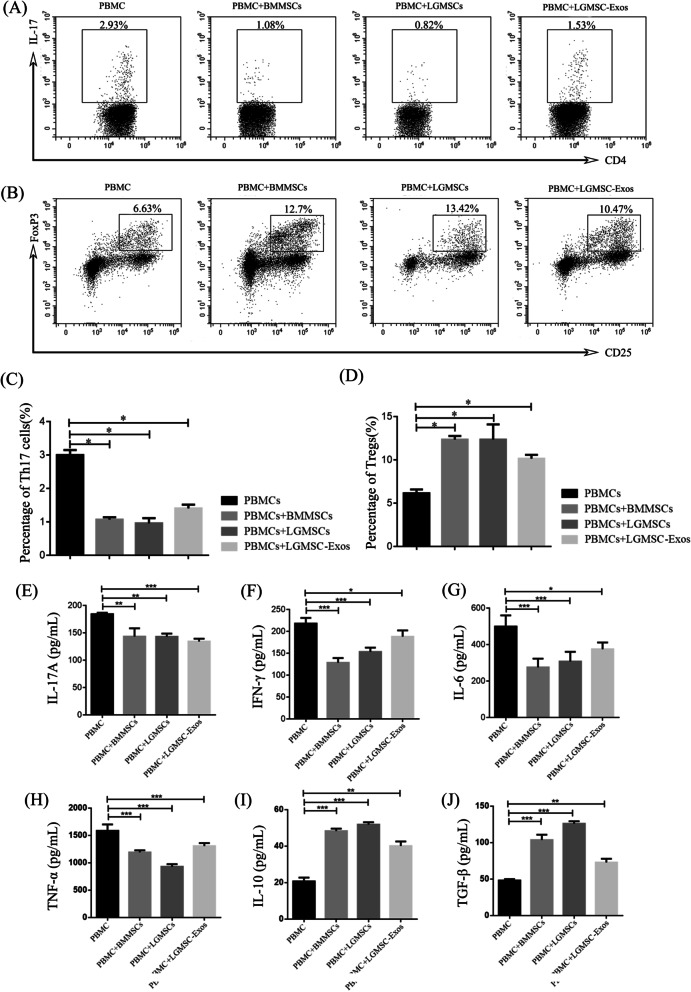
LGMSCs and LGMSC-Exos treatment mediated immune regulation. **A** IL-17^+^ cells in CD4^+^ T cells of untreated and LGMSC- and LGMSC-Exo-treated PBMCs from SS patients. **B** CD25^+^FoxP3^+ ^ cells in CD4^+^ T cells of untreated and LGMSC- and LGMSC-Exo-treated PBMCs from SS patients **C**, **D** Statistics of Th17 and Treg cells. **E**–**J** LGMSCs and LGMSC-Exos regulated the production of T-cell cytokines, namely, IL-17 (**E**), IFN-γ (**F**), IL-6 (**G**), TNF-α (**H**), IL-10 (**I**), and TGF-β (**J**), as analyzed in the supernatant of untreated and LGMSC- and LGMSC-Exo-treated PBMCs from SS patients. Data are shown as mean ± standard deviation (SD) from three independent experiments. ***p < 0.001, **p < 0.01, *p < 0.05. *LGMSCs* labial gland-derived mesenchymal stem cells; *LGMSC-Exos* LGMSC-derived exosomes; *PBMCs* peripheral blood mononuclear lymphocytes; *SS* Sjögren's syndrome; *Th17* T helper 17; *Treg* T regulatory; *IL* interleukin; *IFN-γ* interferon gamma; *TNF-α*: tumor necrosis factor alpha; *TGF-β* transforming growth factor beta

In co-culture system, PKH26-labled LGMSC-Exos were observed in PBMCs (Additional file 1: Figure S1). PBMCs from SS patients were harvested 72 h after co-cultured with LGMSCs and LGMSC-Exos and the percentages of Th17 and Treg cells were estimated. Flow cytometry results showed that the number of Th17 cells was decreased after co-culture with LGMSCs and LGMSC-Exos, whereas the percentage of CD4^+^ CD25^+^ Foxp3^+^ Treg cells was increased after co-culture (Fig. 6A, B).

#### Effects of LGMSCs and LGMSC-Exos on cytokine production in the co-culture system

ELISA was performed to determine whether the immunosuppressive effect of LGMSC and LGMSC-Exos was associated with the regulation of proinflammatory and anti-inflammatory cytokines. The results demonstrated that LGMSCs and LGMSC-Exos treatment significantly suppressed the production of IL-17A, IFN-γ, IL-6, and TNF-α and elevated the production of TGF-β and IL-10 (Fig. 6C–J).

## Discussion

SS is a disease triggered by the breakdown of self–nonself discrimination and a subsequent autoreactive immune response. The majority of SS patients manifest dry eye and dry mouth-related symptoms, which have a severe impact on the quality of life [29]. Current treatment is based on local tear and saliva substitutes, immunosuppressants, and systemic secretagogues, however, these strategies are frequently ineffective and scarcely tolerated [30]. For these reasons, efforts in this field are directed towards developing new potential approaches to inhibit autoreactive immune responses [31]. In recent years, data on the potential applications of MSC therapy for the treatment of autoimmune diseases have progressively increased. The ability of MSCs of different origins of MSCs to modulate the immune response has been well established, and their immunosuppressive activities have been demonstrated in RA, SLE [32–34], and SS [14, 26]. LGMSCs were isolated by our group a few years ago [17], and no studies have explored their possible therapeutic role in SS. In this study, we demonstrated that LGMSCs possess immunoregulatory functions and exhibit evident therapeutic effects in the mouse models of SS. Treatment with LGMSCs decreased the expression of IFN-γ, TNF-α, IL-6, and IL-17, as well as enhanced TGF-β and IL-10 expression and suppressed autoimmune responses, by inducing Treg cells and inhibiting Th17 cells. Although MSCs are considered hypoimmunogenic and overwhelming positive results of MSCs therapy have seen in preclinical animal studies and clinical trials [35–39], the latest opinion about immunogenicity of autologous and allogeneic MSCs has shifted from “immunoprivileged” to MHC-mismatched MSCs may also induce immune responses and are rejected [40]. In this study, we did not find active immune response after treatment. A study once showed that different transplanting routes can lead to divergent immunogenicity of MSCs. MSCs transplanted in normal or in diabetic rats via the tail vein remained immunoprivileged [41]. MSCs transplantion via tail vein is the way we used in the present study. Moreover, Wu J. et al. showed that allo-BMSCs could induce a transient immunoreaction after transplant and their long-term potential therapeutic effect was not affected [42]. The above researches may explain why the immune rejection was not observed  in this experiment.

The immunoregulatory effects of MSCs are believed to be largely mediated by paracrine signals, and several secreted molecules have been identified as contributors. MSCs secrete membrane vesicles, composed of a lipid bilayer including transmembrane proteins and enclosing cytoplasmic components. Such vesicles can transmit signals by interacting at the cell surface, by internalization into endocytic compartments, or by fusion with plasma membranes. In recent years, exosomes released by MSCs have emerged as a novel and effective secretory component of MSCs, and therefore, have gained notable interest. Due to the relatively large cell size, intra-arterial MSC administration may lead to the occlusion of distal vasculature [43]. However, exosomes appear to be much safer for clinical applications due to their relatively small size. Furthermore, exosomes derived from MSCs has not detected MHC I or MHC II complex to date. No explicit immunogenicity of MSC-EXOs has been reported in species crossing studies [44]. MSC-derived exosomes are also more stable and easier to store and transport than MSCs. The application of exosomes has been addressed as exosomes released by MSCs could represent a safer and more reproducible therapeutic tool than their parent cells [21]. In this study, we demonstrated that infusion of LGMSC-Exos significantly improved the saliva flow rate and reduced exocrine gland damage in NOD mice. Further studies demonstrated that LGMSC-Exos possess immunosuppressive functions by regulating CD4 + T cell subsets, increasing the number of Treg cells, and suppressing Th17 cells. Previous studies  have shown that induced pluripotent stem cells and EVs derived from them could prevent SS progression with efficacies comparable to those of BMMSCs and olfactory ecto-MSC-derived exosomes [45, 46]. Although the present results might suggest the effectiveness of LGMSC-derived exosomes in treating SS, what compounds carried by exosomes play the immunomodulatory role is unclear. Exosomes can shuttle various proteins, messenger RNA (mRNA) and microRNAs (miRNAs) to modulate the activity of recipient cells. Future research might focus on exosomal miRNAs as they have been revealed to have the immunologic function and recognized as a key modulator in gene expressions of the immune cells [20].

Although the present study demonstrated that LGMSCs and their exosomes improved the saliva flow rate and reduced exocrine gland damage in NOD mice, the specific therapeutic mechanisms on  salivary gland cells are not clear. According to previous studies, extensive apoptosis of glandular cells has been observed during the development of SS and apoptosis of acinar and ductal epithelial cells has been proposed to be a potential mechanism that impairs the secretion of salivary glands [47–49]. So, we detected if the apoptosis of acinar and ductal epithelial cells were changed after treatment by TUNEL staining. The results reveal that treatment with LGMSCs and LGMSC-Exos significantly reduced apoptotic rate of salivary gland cells (Additional file 1: Figure S3). Future study about how LGMSCs and their exosomes affect apoptosis of salivary gland cells are needed.

Th17 cells produce IL-17, which contributes to the pathogenesis of autoimmune conditions, both in general and specifically in pSS. Accumulating evidence indicates that the proinflammatory cytokine IL-17 is involved in the pathogenesis of SS [50], and the pathogenic role of Th17/IL-17-producing cells in SS were demonstrated by several mouse models of SS [11]. Treg cells play an important role in the maintenance of self-tolerance, and exert suppressive activity on autoreactive lymphocytes. When antigen-driven inflammation persists, the cytokine network appears to favor the propagation of IL-17 producing T cells while minimizing immunosuppressive Treg cells. This imbalance between proinflammatory and anti-inflammatory forces perpetuates the autoimmune lesions, such as in SS. In this study, we demonstrated that treatment with LGMSCs and exosomes released by them corrected this imbalance in the splenocytes of NOD mice and PBMCs from SS patients. However, the possible molecular mechanisms through which LGMSCs and LGMSC-Exos regulate the differentiation of Th17 and Treg subsets requires further exploration.

Upon antigenic stimulation, naïve CD4 + T cells undergo activation, expansion, and differentiation into different effector subsets. Cytokines are produced by Th cells while driving the differentiation of distinct types of Th cells. TGF-β and IL-6 are key cytokines required to induce the Th17 phenotype. Treg cells secrete TGF-β and IL-10 and require the specific cytokine TGF-β and the transcription factor FoxP3 for their differentiation. TGF-β, required for Treg commitment, also participates in the commitment of IL-17-producing Th17 cells. In our study, IL-6 and IL-17 levels were decreased, whereas IL-10 and TGF-β levels were increased in the supernatant of PBMCs from SS patients upon co-culturing the cells with LGMSCs and their exosomes in vitro. These results suggested that the inflammation was inhibited to a certain degree posttreatment.

In summary, we identified the immunoregulatory properties of MSCs from the labial gland and exosomes derived from them in treating SS. LGMSCs and LGMSC-Exos could impart their immunomodulatory activity to ameliorate inflammatory infiltration into exocrine glands and restore salivary gland secretory function in the mouse models of SS. Furthermore, the aforementioned therapeutic effect may be mediated by disrupting the imbalance of Th17/Treg cells. These findings suggest that LGMSCs are effective, and therefore, might be optional MSCs in MSC therapy for SS. Additionally, LGMSC-Exos might be used for developing a novel potential cell-free therapy for inflammatory diseases such as SS.

## Conclusion

This study demonstrated that LGMSCs and LGMSC-Exos could ameliorate inflammatory infiltration into exocrine glands and restore salivary gland secretory function in the mouse models of SS. In addition, it provides insights into the mechanism of LGMSCs or LGMSC-Exos while treating murine SS. LGMSCs and LGMSC-Exos were demonstrated to inhibit the differentiation of Th17 cells but promote the proliferation of Treg cells in vivo and in vitro, accompanied by reduced IL-17, IFN-γ, and IL-6 levels and enhanced secretion of TGF-β and IL-10 by T cells. These findings suggest that LGMSCs might be promising candidates for MSC-based therapy, and LGMSC-Exos might be developed for a new cell-free therapy for SS in future.

## Supplementary Information

**Additional file 1: Figure S1**. LGMSC-Exos uptake by lymphocytes; **Figure S2**. Blood glucose level of NOD mice; **Figure S3**. Apoptosis of salivary gland cells (TUNEL assay).(A) The representative pictures of apoptotic cells of salivary gland cells in NOD mice at each group. (B) Statistical results of apoptosis between groups. NS: not significant compared with NOD+PBS group, ** p < 0.01 compared with NOD+PBS group. HCQ: hydroxychloroquine; LGMSCs: labial gland-derived mesenchymal stem cells; LGMSC-Exos: LGMSC-derived exosomes; DAPI: diamidine phenylindole; TUNEL: terminal-deoxynucleoitidyl transferase mediated nick end labeling; **Table S1**. Basic information of donors of LGMSCs.

## Data Availability

All data generated and/or analysed during this study are included in this published article. Data sharing is not applicable to this article as no datasets were generated or analysed during the current study. However, the data that support the findings of this study are available from the corresponding author upon reasonable request.

## Abbreviations

SS: Sjögren's syndrome
MSCs: Mesenchymal stem cells
LGMSCs: Labial gland-derived MSCs
SLE: Systemic lupus erythematosus
RA: Rheumatoid arthritis
Th: T helper
Treg: Regulatory T
IL-17: Interleukin-17
BMMSCs: Bone marrow-derived MSCs
UCMSCs: Umbilical cord-derived MSCs
EV: Extracellular vesicles
LGMSC-Exos: LGMSC-derived exosomes
PBMCs: Peripheral blood mononuclear cells
PBS: Phosphate-buffered saline
α-MEM: Alpha minimum essential Eagle’s medium
FBS: Fetal bovine serum
TEM: Transmission electron microscopy
NTA: Nanoparticle tracking analysis
HCQ: Hydroxychloroquine
H&E: Hematoxylin and eosin
TGF-β: Transforming growth factor beta
IFN-γ: Interferon gamma
TNF-α: Tumor necrosis factor alpha
SD: Standard deviation
