# Supplementary material for: Labial gland-derived mesenchymal stem cells and their exosomes ameliorate murine Sjögren's syndrome by modulating the balance of Treg and Th17 cells
Source: Stem Cell Res Ther. 2021 Aug 26;12:478. doi: 10.1186/s13287-021-02541-0 (PMC8390194; doi:10.1186/s13287-021-02541-0)
Supplement: Supplementary file 1 — Additional file 1: Figure S1. LGMSC-Exos uptake by lymphocytes; Figure S2. Blood glucose level of NOD mice; Figure S3. Apoptosis of salivary gland cells (TUNEL assay).(A) The representative pictures of apoptotic cells of salivary gland cells in NOD mice at each group. (B) Statistical results of apoptosis between groups. NS: not significant compared with NOD+PBS group, ** p < 0.01 compared with NOD+PBS group. HCQ: hydroxychloroquine; LGMSCs: labial gland-derived mesenchymal stem cells; LGMSC-Exos: LGMSC-derived exosomes; DAPI: diamidine phenylindole; TUNEL: terminal-deoxynucleoitidyl transferase mediated nick end labeling; Table S1. Basic information of donors of LGMSCs. [file 13287_2021_2541_MOESM1_ESM.docx]

Figure S1


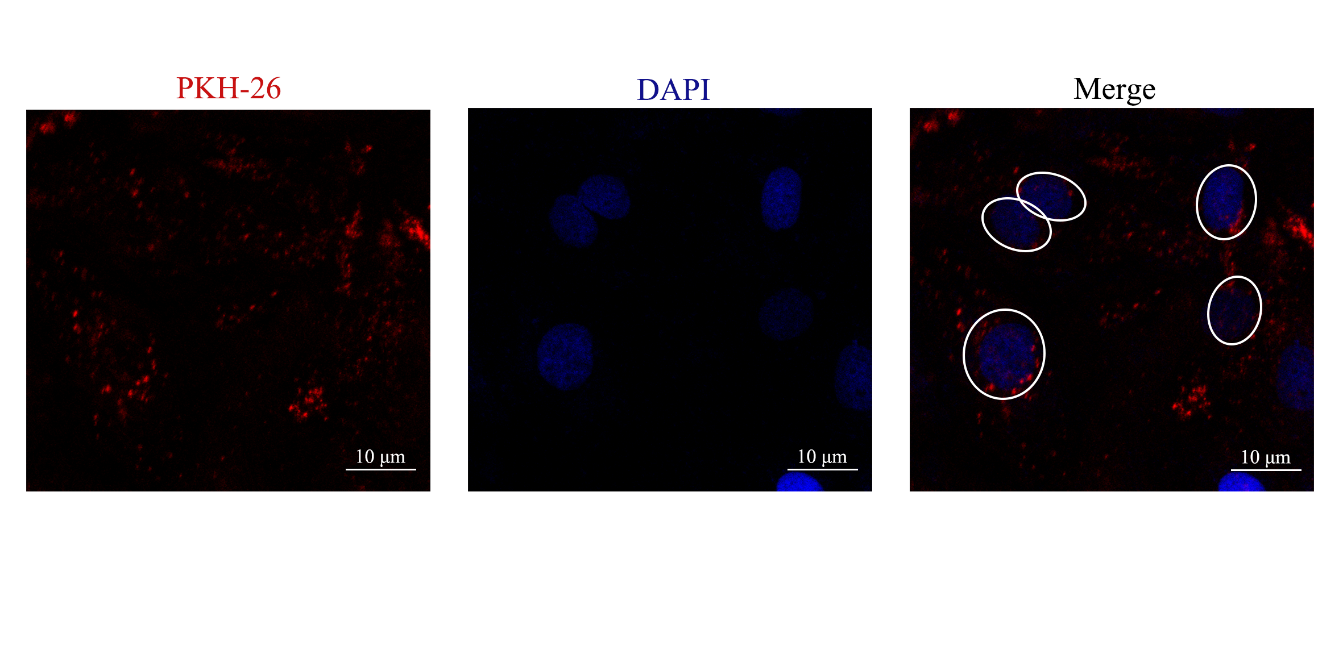


Figure S2


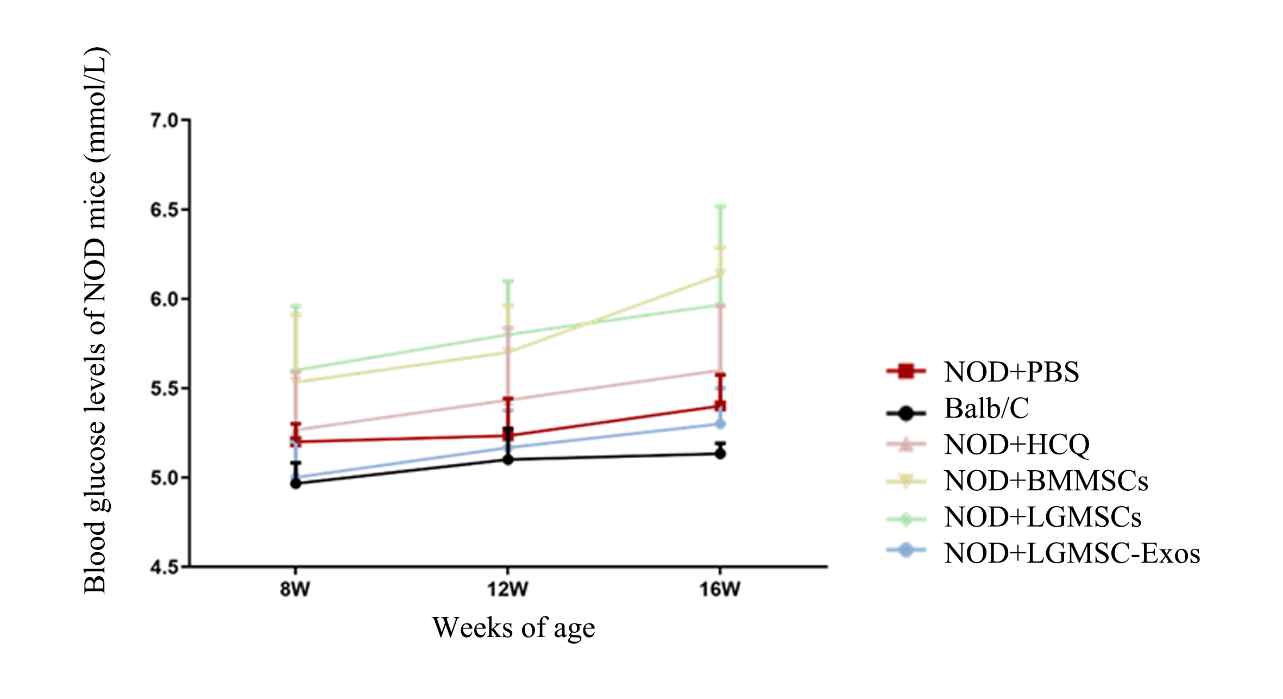


Figure S3


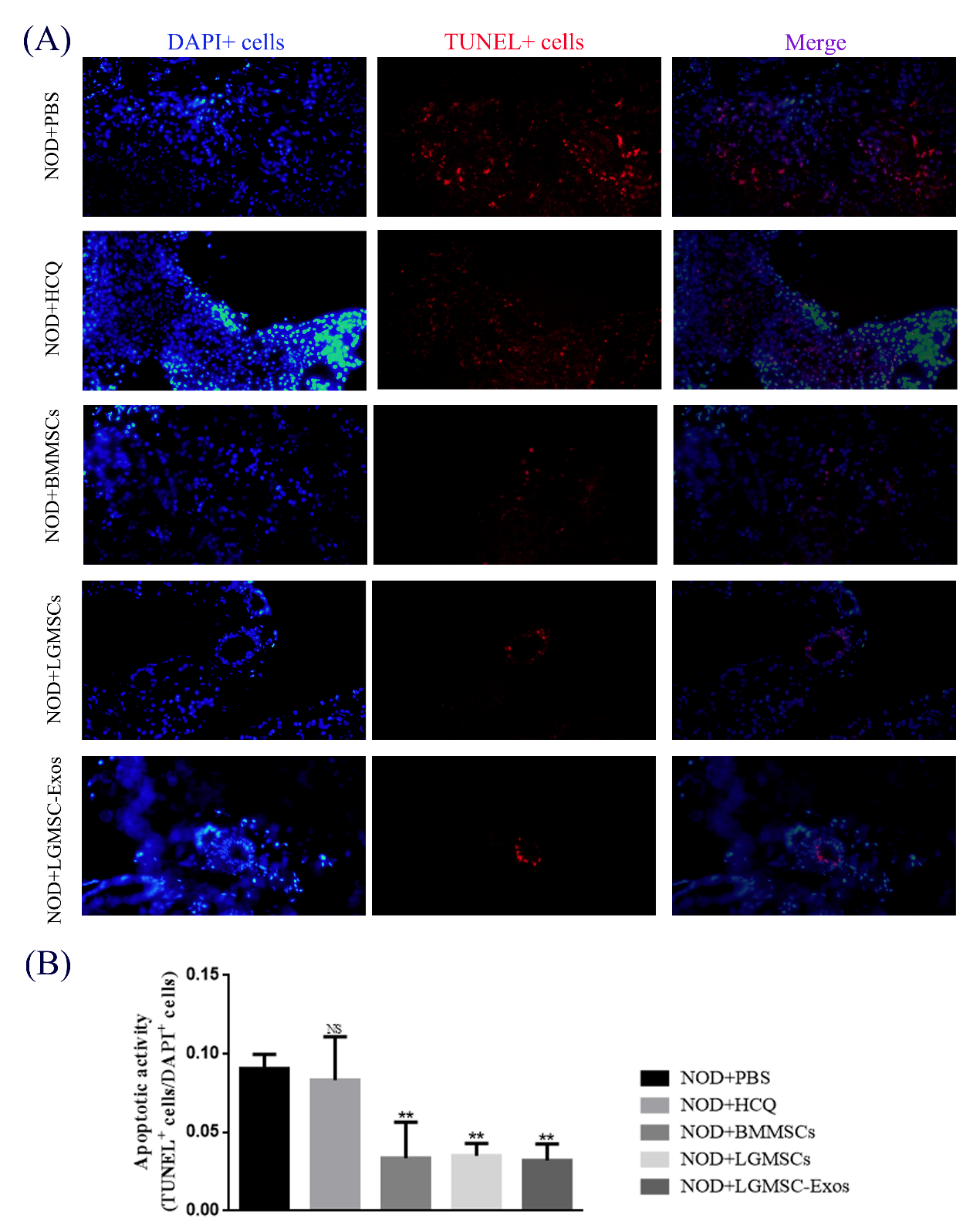


Table S1 Basic information of donors of LG-MSCs

| Donors of labial glands | Sex | Age | Unstimulated salivary flow rate | Head and neck radiation history |
| --- | --- | --- | --- | --- |
| #1 | Female | 32 | 2.1mL/10min | no |
| #2 | Female | 39 | 1.8mL/10min | no |
| #3 | Female | 41 | 1.6mL/10min | no |
| #4 | Male | 30 | 2.5mL/10min | no |
| #5 | Male | 22 | 2.0mL/10min | no |
